# Predicting risk on cardiovascular or cerebrovascular disease based on a physical activity cohort: Results from APAC study

**DOI:** 10.1002/mco2.220

**Published:** 2023-03-10

**Authors:** Juan Zhao, Ye Yu, Xiaolan Zhu, Yuling Xie, Songwei Ai, H. Immo Lehmann, Xuan Deng, Feifei Hu, Guoping Li, Yong Zhou, Junjie Xiao

**Affiliations:** ^1^ Institute of Geriatrics (Shanghai University), Affiliated Nantong Hospital of Shanghai University (The Sixth People's Hospital of Nantong), School of Medicine Shanghai University Nantong China; ^2^ Cardiac Regeneration and Ageing Lab, Institute of Cardiovascular Sciences, Shanghai Engineering Research Center of Organ Repair, School of Life Science Shanghai University Shanghai China; ^3^ School of Pharmacy Shanghai University of Traditional Chinese Medicine Shanghai China; ^4^ Clinical Research Institute, Shanghai General Hospital Shanghai Jiaotong University School of Medicine Shanghai China; ^5^ Cardiovascular Division of the Massachusetts General Hospital and Harvard Medical School Boston Massachusetts USA

**Keywords:** cardiovascular disease, cerebrovascular disease, cohort study, cox proportional hazard regression, physical activity

## Abstract

Commonly used prediction models have been primarily constructed without taking physical activity into account. Using the Kailuan physical activity cohorts from Asymptomatic Polyvascular Abnormalities in Community (APAC) study, we developed a 9‐year cardiovascular or cerebrovascular disease (CVD) risk prediction equation. Participants in this study were included from APAC cohort, which included 5440 participants from the Kailuan cohort in China. Cox proportional hazard regression model was applied to construct sex‐specific risk prediction equations for the physical activity cohort (PA equation). Proposed equations were compared with the 10‐year risk prediction model, which is developed for atherosclerotic cardiovascular disease risk in Chinese cohorts (China‐PAR equation). *C* statistics of PA equations were 0.755 (95% confidence interval, 0.750–0.758) for men and 0.801 (95% confidence interval, 0.790–0.813) for women. The estimated area under the receiver operating characteristic curves in the validation set shows that the PA equations perform as good as the China‐PAR. From calibration among four categories of predicted risks, the predicted risk rates by PA equations were almost identical to the Kaplan–Meier observed rates. Therefore, our developed sex‐specific PA equations have effective performance for predicting CVD for physically active cohorts in the physical activity cohort in Kailuan.

## Graphical abstract

This study used the participants from the Asymptomatic Polyvascular Abnormalities in Community (APAC) to develop a new sex‐specific prediction equation that involves physical activity (PA equation) for cardiovascular (CVD) risk prediction. The developed sex‐specific PA equations have shown good performance to predict CVD for a physical active cohort in the Kailuan population that will help to improve the prevention and treatment of CVD.

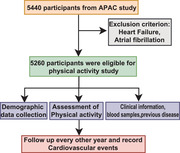

## 1 INTRODUCTION

Cardiovascular or cerebrovascular diseases (CVD) remain the leading cause of mortality for people in China and many other countries around the world, including atherosclerotic cardiovascular disease (ASCVD), heart failure (HF), atrial fibrillation (AF), myocardial infarction (MI), and stroke.^1^, ^2^ According to the survey from 2015 to 2018 by National Health and Nutrition Examination, about 6 million Americans over the age of 20 years had HF with a fairly high number of black males and females.^2^ In China, CVD is increasingly threatening people's health^2^; this is lightened by the rapid economic development and the change in lifestyle over the past three decades. Besides, CVD mortality is significantly higher in the population of Northern China compared with Southern China.^3^, ^4^ Although CVD mortality is as serious as the mortality of malignancies, it commonly can be prevented or well controlled by early interventions.^5^ Hence, early warning and identification of subjects at risk is pertinent for preventing and treating of CVD.

Many risks prediction models and equations have been put forward and shown to have high performance in different populations.^6^, ^7^, ^8^, ^9^ Well‐known prediction models for Western populations include the Framingham risk equations for CVD, which is developed based on the populations in America^10^ and the Pooled Cohort Equations for estimating the10‐year risk of ASCVD for White and African American populations.^11^ For United Kingdom, the cardiovascular disease risk algorithm (QRISK) has been established as a new 10‐year CVD risk score in the derivation cohort.^12^ For Asia, Barzi et al.^9^ developed a cardiovascular risk prediction tool. Chien et al.^13^ constructed a 15‐year prediction model for predicting incidence of stroke in Chinese adults. The China‐PAR project established the Chinese ASCVD risk estimation tool (Prediction for ASCVD Risk in China).^14^ However, theses prediction models all derive from cohorts that did not take the physical activity of participants into account.

Physical activity represents a cornerstone in primary level of prevention for CVD, compared with participants with no physical activity, even low intensity of physical activity was correlated with the decreased risk of mortality.^2^ It has been shown to have protective effects in several tissues; in the brain, it has been shown to be positively associated with the total brain volume and cortical thickness in temporal areas such.^15^ It has been shown to reduce mortality and morbidity of CVD and importantly, intensity of physical activity appears be beneficial for female life expectancy.^16^ Increasing time spent in low‐intensity physical activity protects adolescents from effects of long‐term sedentary lifestyle.^17^, ^18^ Intervention measures are needed to promote regular physical activity in school to decrease the sedentary time for child and teenager.^19^ Study also shows that moderate physical activity was inversely associated with coronary heart disease.^20^, ^21^ Various evidence has shown that physically active subjects have a lower incidence of CHD and CVD.^22^ Thus, physical activity epidemiology has even been born under this circumstance.^22^ However, in previous risk prediction models, physical activity has not been considered as a factor.^14^, ^23^ Therefore, it is critical to establish new tools for prediction of CVD that includes physical activity.

**FIGURE 1:**
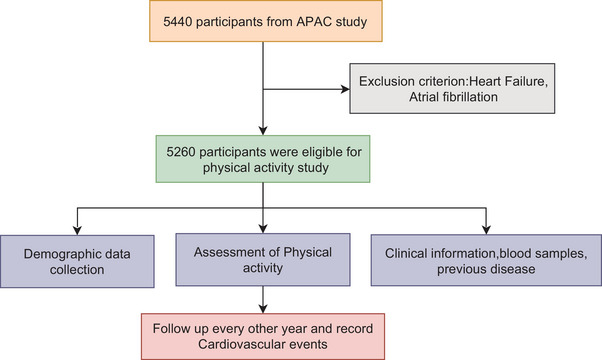
Flow chart of data collection. Participants in this study were included from Asymptomatic Polyvascular Abnormalities in Community (APAC) cohort, which included 5440 participants from the Kailuan study in China. Demographic data and information on physical activity were collected by questionnaires. Exclusion criteria were previous heart failure (HF) or atrial fibrillation (AF).

The Asymptomatic Polyvascular Abnormalities in Community (APAC) cohort as a population‐based study was designed to verify the prevalence and correlations of symptomatic polyvascular abnormalities in the ordinary population.^24^ The study includes 5440 participants with 40.1% women with age over 40 years. The APAC evaluated the correlation between retinal nerve fiber layer changes, retinal microvascular signs, intracranial arterial stenosis, and eventually study the occurrence and development of a cardiovascular or cerebrovascular event. In the APAC study, assessment for the intensity of physical activity were conducted based on the International Physical Activity Questionnaire (IPQA) short form for all participants^25^, ^26^, ^27^ under the instruction of professional and well‐trained researchers. The Kailuan physical activity cohorts used in our study were created from the individuals in APAC study by excluding those who have previously reported HF or AF, and the details can be found in flow chart in Figure 1. Hence, in this study, we first used the participants from the APAC study,^24^ to develop a new prediction equation that involves physical activity (PA equation) for prediction of CVD events. Second, we validated our PA equation by a 10‐fold cross validation and compared it with the China‐PAR project by estimating the area under the receiver operating characteristic (ROC) curves (AUC).

## 2 RESULTS

### 2.1 Baseline characteristics of participants by sex

The baseline characteristics of participants were presented in Table 1, and the *p* value less than 0.05 represents the significant difference of characteristics between men and women. The average age of the 5260 participants was 55.0±11.70 years, and 60% of participants was men. Overall, men (56.9 ± 12.6 years) were older on average than women (52.1 ± 9.6 years) (*p* value < 0.001). For education, most of participants had completed secondary school or higher. 51.4% women attended high school or above, while only 39% of men did. About half of male participants were current smokers. In contrast, females were less likely to be current smokers, only 1.8% (*p* value < 0.001). Similarly, men (23.8%) were more likely to be current drinkers than women (0.3%). Men also on average had higher diastolic blood pressure (DBP) and systolic blood pressure (SBP) than women (*p* value < 0.01). Besides, men were more likely to have a history of hypertension than women with *p* value = 0.005. The statistical results of baseline characteristics of participants by sex suggested the significant difference between men and women for most of the basic demographic variables such age, education, SBP as well as the past medical history.

**TABLE 1:** Baseline characteristics of participants in the physical activity cohort.

Characteristics	Total (*N* = 5260)	Male (*N* = 3135)	Female (*N* = 2125)	*p* Value
Age, mean (SD), y	55.0±11.70	56.9±12.6	52.1±9.6	<0.001
Education, *n* (%)				
Primary school	623 (11.8)	449 (14)	174 (8.2)	<0.001
Secondary school	2323 (44.1)	1464 (47)	859 (40.4)	
High school or above	2314 (44.0)	1222 (39)	1092 (51.4)	
BMI, mean (SD), kg/m^2^	24.8±3.1	25.1±3.2	23.3±3.5	<0.001
Current smoker, *n* (%)	1674 (31.8)	1636 (52.2)	38 (1.8)	<0.001
Current drinker, *n* (%)	754 (14.3)	746 (23.8)	8 (0.3)	<0.001
SBP, mean (SD), mmHg	131.0±20.0	134.6±19.0	125.8±20.2	<0.001
DBP, mean (SD), mmHg	82.8±11.1	84.9±10.8	79.3±10.8	<0.001
Previous history of disease, *n* (%)				
Hypertension	1326 (25.2)	843 (26.9)	483 (22.7)	<0.001
Diabetes	400 (7.6)	245 (7.8)	155 (7.3)	0.48
Hyperlipemia	578 (11.0)	297 (9.5)	281 (13.2)	<0.001

*Note*: Categorical variables were represented as number (percentage); continuous variables were represented as mean ± standard deviation(SD). BMI, body mass index; SBP, systolic blood pressure; DBP, diastolic blood pressure. The *p* value < 0.05 indicates the significant difference of characteristics between men and women.

### 2.2 Baseline characteristics of participants by physical activity

Baseline characteristics of participants based on physical activity were presented in Table 2, and the *p* value less than 0.05 represents the significant difference of characteristics among three levels intensity of physical activity groups. As described above, we classified subjects into 3 classes based on the intensity of physical activity. 40%, 25% and 35% subjects had low‐intensity activity, moderate‐activity and high‐intensity activity levels, respectively. Subjects with high‐intensity activity were older than moderate and low‐intensity activity (*p* value < 0.001). Low‐intensity activity subjects were more likely to be current smoker and drinker (*p* value < 0.001).

**TABLE 2:** Baseline characteristics based on intensity of physical activity.

Characteristics	Total (*N* = 5260)	Low intensity (*N* = 2104)	Moderate intensity (*N* = 1339)	High intensity (*N* = 1817)	*p* Value
Sex (Men/Women)	3135/2125	1301/803	701/638	1133/684	<0.001^a^, ^c^
Age, mean (SD), y	55.0±11.7	52.6±10.7	53.0±11	59.2±12.2	<0.001^b^, ^c^
Education, *n* (%)					
Primary school	623 (11.8)	275 (13.1)	118 (8.8)	230 (12.7)	<0.001^a^, ^b^, ^c^
Secondary school	2323 (44.1)	1039 (49.4)	522 (39.0)	762 (41.9)	
High school or above	2314 (44.0)	790 (37.5)	699 (52.2)	825 (45.4)	
BMI, mean (SD), kg/m^2^	24.9±3.3	25.0±3.4	25.0±3.2	24.8±3.1	0.025^b^, ^c^
Current smoker, *n* (%)	1674 (31.8)	819 (38.9)	365 (27.3)	490 (27.0)	<0.001^a^, ^b^
Current drinker, *n* (%)	754 (14.3)	359 (17.1)	169 (12.6)	226 (12.4)	<0.001^a^, ^b^
SBP, mean (SD), mmHg	131.0±20.0	130.2±19.1	129.1±19.6	133.4±20.9	<0.001^b^, ^c^
DBP, mean (SD), mmHg	82.8±11.1	82.3±11.2	82.7±10.9	82.5±11.0	0.055
History of disease, *n* (%)					
Hypertension	1326 (25.2)	499 (23.7)	312 (23.3)	515 (28.3)	<0.001^b^, ^c^
Diabetes	400 (7.6)	137 (6.5)	83 (6.2)	180 (9.9)	<0.001^b^, ^c^
Hyperlipidemia	578 (11.0)	192 (9.1)	162 (12.1)	224 (12.3)	0.001^a^, ^b^

*Note*: Categorical variables were represented as number (percentage); continuous variables were represented as mean ± standard deviation (SD).

BMI, body mass index; SBP, systolic blood pressure; DBP, diastolic blood pressure.

The *p* value is the results of overall comparisons, where *p* value < 0.05 indicates the significant difference of characteristics among the intensity of three groups, that is at least two groups are significantly different. The superscript indicates the detailed significant groups under significant level 0.05, where:

^a^ represents low versus moderate group.

^b^ represents low versus high group.

^c^ represents moderate versus high group.

**FIGURE 2:**
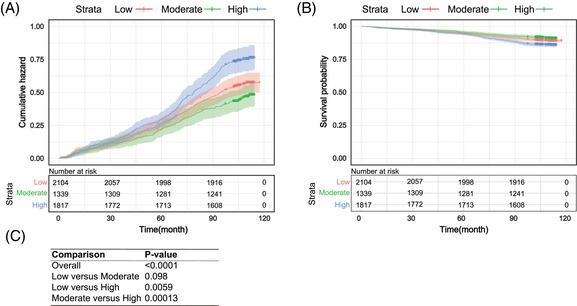
Comparison of Kaplan–Meier survival probability and cumulative hazard curves for three intensity of physical activity group; (A) the cumulative hazard curve for three groups of physical activity intensity. The vertical axis represents the cumulative hazard; (B) the comparison of survival time for three groups, the vertical axis is the survival probabilities for three levels of physical activity intensity. The red, green, and blue line represent the results of low‐intensity group, moderate‐intensity group, and high‐intensity group respectively in both A and B. The confidence intervals were presented in the figures. (C) The results of *p* values for overall comparison and pairwise comparison for both A and B, where *p* value < 0.05 indicated that there were significant difference between groups.

Based on Kaplan–Meier survival analysis results, the KM‐survival curve was significantly different among groups of physical activity intensity, as shown in Figure 2A. And the cumulative hazard in Figure 2B also significantly different between three intensity of physical activity groups. The *p* value for the overall and pairwise comparison were calculated by using log‐rank test, and the results can be found in Figure 2C. According to the *p* values of pairwise comparison, we can find that Figures 2A and B also indicated that the moderate intensity of physical activity group had the longest median survival time, meanwhile it has the lowest hazard ratio compared with the other two groups at the same time. The cumulative hazard ratio of the high‐intensity physical activity group is statistical significantly higher than other two groups, and its survival time is significantly less than the other two groups. The survival time and the cumulative hazard rates between the low and moderate groups were not significantly different. The results of baseline characteristics of participants by physical activity suggested importance of physical activity in the study of CVD events.

### 2.3 PA equation training

Sex‐specific physical activity equations (PA equations) were developed for predicting 9‐year CVD risk based on risk factors which included age, total cholesterol (CHOL), high‐density lipoprotein cholesterol (HDLC), SBP, current smoking state, diabetes mellitus, and physical activity. A Cox proportional hazard regression model was used to construct the prediction model for CVD in the different groups of PA. Details of parameters were shown in Table 3. Values of the parameters were the mean from 10‐fold cross validations. We found that physical activity was inversely associated with hazard rate of CVD. In the training cohort, we used discrimination *C* statistic as well as calibration *χ* ^2^ to validate PA equations. Results of *C* statistic and calibration *χ* ^2^ showed that women had higher value of *C* statistic and calibration *χ* ^2^ numerically than men (0.801 vs. 0.755). Calibration *χ* ^2^ values for both men and women were very high which indicated the significance of the PA equation (Table 4). In general, the results indicate PA equations have high *C* statistic and calibration *χ* ^2^ in men and women in the training set. Hence, we can use the PA equations for predictions in the validation cohorts.

**TABLE 3:** Sex‐specific parameters of equations for predicting 9‐year CVD risks.

	Men		Women	
	Coefficient	*p* Value	Coefficient	*p* Value
Age	35.46	<0.001	21.24	0.32
CHOL	2.97	0.67	−0.46	0.68
HDLC	−5.8	0.02	−0.46	0.05
SBP	27.12	<0.001	15.80	0.03
Smoke	5.74	0.007	2.62	0.98
Diabetes	0.33	0.02	0.79	<0.001
Physical activity	−0.12	0.03	−0.03	0.05
Age:SBP	−6.11	<0.001	−3.38	0.03
Age:Smoke	−1.29	0.013	−0.67	0.97
Age:CHOL	−0.68	0.57	NA	0.39
Age:HDLC	1.28	0.15	NA	0.06
Mean(coefficient × value)	155.04		79.05	
Baseline survival rate	0.90		0.97	

*Note*: CHOL, total cholesterol; HDLC, high‐density lipoprotein cholesterol; SBP, systolic blood pressure; Age:X represents the interaction term between age and X. NA means not included in the model. *p* Value < 0.05 indicates the variables were significant in the model. PA Equation: risk = 1 − *S*_10_^(indSum − Mean)^ where indSum is coefficient × individual values, and Mean is average of coefficient × individual values of all subjects. *S*_10_ is baseline survival rate.

**TABLE 4:** 10 × 10 cross‐validation for training sets.

	Men (*N* = 3135)		Women (*N* = 2125)	
	*C* statistics	Calibration *χ* ^2^	*C* statistics	Calibration *χ* ^2^
Original value	0.755	328.5	0.8	142.7
Mean	0.76	296.8	0.8	137
SD	0.003	7.77	−0.46	5.99
95% CI	0.75–0.76	286.9–307.6	0.007	122.6–130.7
Median	0.76	296.2	0.79–0.81	130

*Note*: SD means the standard deviation. The 95%CI represents the 95% confidence interval of each statistic.

### 2.4 PA equation validation

**FIGURE 3:**
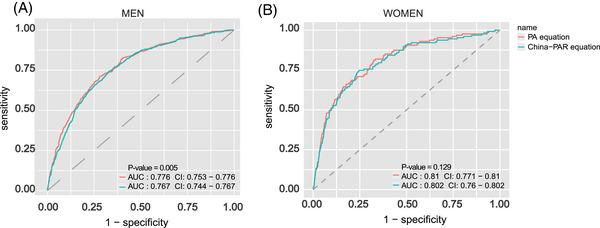
Comparison the Receiver operating characteristic (ROC) curves for men and women between PA equations and China‐PAR equations. China‐PAR was the abbreviation for “Prediction for ASCVD Risk in China” model. A and B were the results in men and women, respectively. The pink line represented the results from PA equations and the green line was the results of China‐PAR equation. The values of AUC and *p* value were shown on bottom right corner. *p* Values were the comparison between PA equation and China‐PAR equation in men and women, respectively.

**FIGURE 4:**
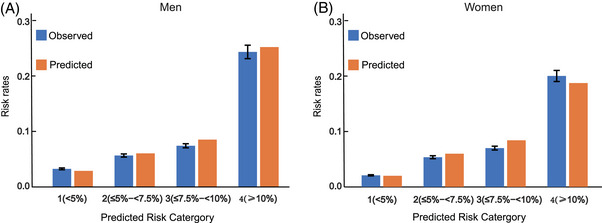
Kaplan–Meier observed and predicted CVD event rates using PA equations. A and B were observed and predicted event rates in men and women respectively. The blue bars were observed risks and the orange ones were the predicted CVD events rates calculated from PA equations. The *X*‐axes is the risk categories, and the *Y*‐axes is the probability of CVD event. The predicted risk categories were labeled by 1, 2, 3, and 4, where 1 represents risk rates are less than 5% (<5%), 2 represents risk rates are more than or equal to 5% and less than 7.5% (≤5−<7.5%), 3 represents the risk rates are more than or equal to 7.5% and less than 10% (≤7.5−<10%), and 4 represent the risk rates are more than or equal to 10% (≥10%).

In the validation cohort, we compared the PA equation with the China‐PAR equation. As shown in Figures 3A and B, the AUCs for the PA equations were as good as China‐PAR in both men and women, in particular, the AUC by PA equations was significantly higher than China‐PAR in men (*p* value = 0.005) (Figure 3A). This result indicated that the PA equations were better fitted than the China‐PAR in the physical activity cohort for Men. In addition, to further validate performance of PA equations, we divided participants into four categories (<5%, ≤5−<7.5%, ≤7.5−<10%, and ≥10%) based on the predicted risks. Categories were verified clinically meaningful cut points.^14^ Figure 4 were the comparison between Kaplan–Meier observed and predicted CVD event rates using PA equations. Histogram showed that predicted risk ratios were very close to observed risks in each cluster in both men and women. (Figures 4A and B). Overall, the results of AUCs and the histogram of predicted risks underscore the accuracy of our method, and the validation results also shown that compared with China‐PAR model, our sex‐specific PA equations were more applicable to the physical activity cohorts, especially in men.

## 3 DISCUSSION

This study investigated the physical activity cohort from Kailuan, China. Major findings of the present work are as follows. First, we found that physical activity was significantly associated with positive cardiovascular health outcome. Subjects who conducted moderate intensity of physical activity have the lower hazard ratio for CVD at the same time points comparing with the low and high group. On the contrary, the moderate group has longer survival time than the other two groups. Second, we developed physical activity equations (PA equations) for the Kailuan physical activity cohorts. From the PA equations, we found that physical activity reduced the hazards ratios of CVD.

The primary difference between our PA equations and prior published equations is, that we included physical activity into the risk estimating equation. Variables used in our model were age, SBP, CHOL, HDLC, current smoking state (yes/no), diabetes mellitus (yes/no), and physical activity. Of note, these variables are similar to those that have been used in other prediction models, except for the factor of physical activity. The values of the parameters in PA equations were close to China‐PAR equations, which were developed based on the Chinese population. Our data show that PA equations perform better in physically active cohorts than China‐PAR for Men.

In our study, physical activity was assessed using an international questionnaire short form, which is slightly different from the Harvard Alumni Questionnaire^16^, ^28^ and other physical activity questionnaires.^29^ We classified subjects into three classes based on amount of physical activity times and frequencies per week. The amount of physical activity influenced risk prediction of CVD in this cohort. Previous studies have shown an association between the intensity of physical activity and the risk for the development of heart disease and CVD.^30^, ^31^ The American Heart Association and the American College of Cardiology give the recommendation to adults to engage into moderate‐intensity physical activity around 150 min per week of or high‐intensity physical activity around minutes per week.^11^ The 2018 American Physical Activity Guidelines also provide an amount of physical activity for people of different age groups.^32^ In China, studies about the amount of physical activity have been scare, and in 2018, Shanghai Children's Medical Center released the first Physical activity guidelines for Chinese children and adolescents.^33^ Hence, our study on the association between CVD risk and the amount of physical activity provides valuable guidance for Chinese patients regarding primary prevention of CVD.

Several limitations and deficiencies of the present study should be highlighted. First, in this work, we only performed internal cross validation without external validation of PA equation because of the limitation of cohort. Hence, we need to be careful when applying the PA equations to other populations. Therefore, in future studies, we will further validate PA equations in more physical activity cohorts. Second, the use of the Kailuan population limits this study; currently, the participants were only followed‐up 9 years on average, and the size of subjects used in this study was not very big, particularly in a primary prevention trial with low outcome rates. The size may also have effect on the bias of the prediction model.^7^ Third, in this study, we used “IPQA” to measure physical activity levels, and it will be important to add objective criteria such as objectively measured physical activity by using accelerometer in the future. Last but not the least, during the 9‐year follow‐up, the intensity of physical activity may have changed for each participant and thus, physical activity can be a dynamic variable that changes over time. In our model, we did not account for changes in the physical activity and the subsequent risk of CVD. Besides, it should be stressed that the PA equation is better in men than women. From Table 3, it is obvious that most of the factors’ coefficients of PA equation were significant in men. In contrast, several factors’ coefficients were not significant in women. There could be many reasons for this, such as the gender difference in CVD.^34^ It has been documented that age, CHOL and HDLC have strong effects in men, but diabetes and smoking have main influence in women. However, the baseline characteristics in Table 1 showed that the percentage of women who smoke is very low in our cohort. Hence, the equation for predicting risk of CVD might not be as robust or reliable in female population. In addition, the intensity of physical activity was significantly different between men and women. The number of women with different intensity of physical activity was relatively balanced (as shown in Table 2). By contrast, the proportion of men with different intensity of physical activity was various (as shown in Table 2). Nevertheless, we developed PA equations for physical activity cohorts, which provides an insight on the development of prediction models for CVD, which will have clinical values in the future.

In conclusion, we developed a prediction model for a physically active cohort based on a Cox proportional hazard regression. Our developed sex‐specific PA equations have shown good performance to predict CVD for a physically active cohort in the Kailuan population. The results suggest that physical activity has an inverse association with CVD risk in this population.

## 4 METHODS

### 4.1 Ethics statement

The study was performed on the basis of the guideline of the Declaration of Helsinki and was approved by the Ethics Committees of the Kailuan General Hospital, Beijing Tongren Hospital, and Beijing Tiantan Hospital. The study was also approved by the Ethics Committee of Shanghai University (2021‐195). We obtained written consent from all participants at the time of study inclusion.

### 4.2 The sources of participants

The participants in this study were ≥18 years old and agreed to participate in follow‐up from Jidong community, Caifeidian District, Tangshan city in Hebei province in China.^23^ The participants were followed up once every other year from 2011 to 2019, a relatively long time span. The Kailuan group is China's large coal mining enterprises, which includes 101,510 employees and retirees. The study cohort of this work was selected from APAC study with 5440 participants. The APAC study is a subpopulation of the Kailuan cohort, which were obtained by adopting stratified random sampling based on age and sex according to the 2010 China National Population Census, the main inclusion criteria for APAC study were no history of stroke, coronary disease and transient ischemic attack.

### 4.3 Study design and study participants

In this study, we used participants from the previously published “APAC” cohort, a subpopulation of the Kailuan study. The APAC exclusion criteria were previous reported history of stroke, transient ischemic attack, and coronary disease at baseline and absence of neurologic deficits for stroke.

In this study, we further excluded subjects with a previously reported HF or AF. One hundred eighty subjects were excluded because of lack of information or a history of CVD. Finally, 5260 participants were included to develop and validate the sex‐specific physical activity equations (PA equations) (the detailed flow chart can be found in Figure 1).

A baseline examination was conducted at the time of study inclusion. Basic demographic variables such as information of lifestyle and education as well as the past medical history were collected with standardized questionnaires. Body weight and height were measured, and body mass index was calculated as kilograms per meters squared. Information on smoking, drinking, and physical activity were also collected by questionnaires. SBP and DBP were taken at a 5‐min interval, values were measured twice and the mean of the SBPs and DBPs was respectively used.

Physical activity was recorded by using the standardized IPAQ short form under the assistance of professional researchers. The subjects were asked about the time and frequencies of exercises. We then divided the physical activity into low‐intensity activity (<10 min/week), moderate‐activity (10–80 min/week), and high‐intensity activity (>80 min/week). The first follow‐up was conducted between 2011 and 2012. Individuals were followed up every 2 years through face‐to‐face interviews. Subjects with a history of CVD diseases, including MI, chronic ischemic (CI) HF, cerebral hemorrhage (CH), and stroke were excluded.

### 4.4 Classification of events

The participants were followed up once every year and the events were collected during the follow‐up periods from 2011 to 2019. The events were classified based on the first occurrence of CVD. The primary outcomes of CVD included MI, CI HF, CH, and all‐cause mortality. In this study, we combined the total events of MI, CI, CH, and all‐cause mortality as CVD events in the prediction model.

### 4.5 Statistical analysis

All the statistical data analyses were performed in R with version 4.0.3 (2020‐10‐10). Kolmogorov–Smirnoff test was used to perform normality test for continuous variables. For the normally distributed variables, we used Student's *t*‐test and one‐way ANOVA to compare among different groups, otherwise nonparametric method Wilcoxon test was used for the variables that do not follow normal distribution. For categorical variables, the χ^2^ test was used to compare the difference among groups. *p* Value < 0.05 indicates there were significant differences among the groups.

Sex‐specific Cox proportional hazard regression^35^, ^36^, ^37^ was used to construct a risk prediction model to predict the probability of developing CVD over a 9‐year follow up. In order to maximize performance of the calibration and discrimination for the prediction models and to minimize the impact of extreme observations, natural log–transformation for all continuous covariates in the model was performed. Risk factors in our models were selected by using the traditional cardiovascular risk factors including age, current smoking state (yes/no), CHOL, HDLC, and diabetes.^38^ In addition to these factors, we also include the SBP and physical activity in our model. Hence, the selected factors were the same as China‐PAR^14^ in addition to physical activity. Besides, the interaction terms between age and other risk factors were also included in the model. Participants were randomly divided into training cohort and validation cohort based on the R function “createFolds” in the R package caret.^39^ Ten‐fold cross validation was performed for the internal validation. *C* statistic, calibration *χ* ^2^ were calculated to evaluate the performance of the equations.^40^, ^41^, ^42^ PA equations were also compared with the China‐PAR equation^14^ by the ROC curves. We also calculated the AUC for quantitative comparison; the log‐rank test was used to calculate *p*‐value for checking whether the difference was significant.^43^

## AUTHOR CONTRIBUTION

J. X. and Y. Z. conceived the protocol. J. Z., Y. Y., X. Z., Y. X., S. A., X. D., and F. H. contributed to the analysis and interpretation of data. J. Z. drafted the manuscript. J. X., Y. Z., H. L., and G. L. critically revised the manuscript. All authors agree to be fully accountable for ensuring the integrity and accuracy of the work and read and approved the final manuscript. The corresponding author had full access to all data in the study and assumed final responsibility for the decision to submit the manuscript for publication.

## CONFLICT OF INTEREST STATEMENT

The authors declare no competing interest.

## ETHICS STATEMENT

The study was conducted on the basis of the guideline of the Declaration of Helsinki and was approved by the Ethics Committees of the Kailuan General Hospital, Beijing Tongren Hospital, and Beijing Tiantan Hospital. The study was also approved by the Ethics Committee of Shanghai University (2021‐195). Written informed consent was taken from all participants at the time of study inclusion.

## Data Availability

The datasets generated for this study are available on request to the corresponding author upon reasonable request.
